# Inside-out, antimicrobial resistance mediated by efflux pumps in clinical strains of *Acinetobacter baumannii* isolated from burn wound infections

**DOI:** 10.1007/s42770-024-01461-4

**Published:** 2024-07-24

**Authors:** Melissa Hernández-Durán, Claudia Adriana Colín-Castro, Diana Fernández-Rodríguez, Gabriela Delgado, Rosario Morales-Espinosa, María Guadalupe Martínez-Zavaleta, Chandra Shekhar, Jossue Ortíz-Álvarez, Rodolfo García-Contreras, Rafael Franco-Cendejas, Luis Esaú López-Jácome

**Affiliations:** 1 Laboratorio de Microbiología Clínica, División de Infectología, Instituto Nacional de Rehabilitación Luis Guillermo Ibarra Ibarra, Mexico City, Mexico; 2 Plan de Estudios Combinados en Medicina (PECEM) MD/PhD, Facultad de Medicina, Universidad Nacional Autónoma de México, Mexico City, Mexico; 3 Laboratorio de Genómica Bacteriana, Departamento de Microbiología y Parasitología, Facultad de Medicina, Universidad Nacional Autónoma de México, Mexico City, Mexico; 4 College of Medicine, The University of Tennessee Health Science Center, Memphis, USA; 5 Ciencias y Tecnologías (CONAHCYT), Programa “Investigadoras E Investigadores Por México”. Consejo Nacional de Humanidades, Mexico City, Mexico; 6 Laboratorio de Bacteriología, Departamento de Microbiología y Parasitología, Facultad de Medicina, Universidad Nacional Autónoma de México, Mexico City, Mexico; 7 Biomedical Research Subdirection, Instituto Nacional de Rehabilitación Luis Guillermo Ibarra Ibarra, Mexico City, Mexico; 8 Departamento de Biología. Facultad de Química, Universidad Nacional Autónoma de México, Mexico City, Mexico

**Keywords:** *Acinetobacter baumannii*, Antibiotic resistance, Efflux pumps, Clinical strains, Burn wound infection

## Abstract

**Abstract:**

*Acinetobacter baumannii* belongs to the ESKAPE group. It is classified as a critical priority group by the World Health Organization and a global concern on account of its capacity to acquire and develop resistance mechanisms to multiple antibiotics. Data from the United States indicates 500 deaths annually. Resistance mechanisms of this bacterium include enzymatic pathways such as ß-lactamases, carbapenemases, and aminoglycoside-modifying enzymes, decreased permeability, and overexpression of efflux pumps. *A. baumannii* has been demonstrated to possess efflux pumps, which are classified as members of the MATE family, RND and MFS superfamilies, and SMR transporters. The aim of our work was to assess the distribution of efflux pumps and their regulatory gene expression in clinical strains of *A. baumannii* isolated from burned patients.

**Methods:**

From the Clinical Microbiology Laboratory at the Instituto Nacional de Rehabilitación Luis Guillermo Ibarra Ibarra collection in Mexico, 199 strains were selected. Antibiotics susceptibilities were performed by broth microdilutions to determine minimal inhibitory concentrations. Phenotypic assays with efflux pump inhibitors were conducted using carbonyl cyanide 3-chlorophenylhydrazone (CCCP) and phenylalanine-arginine ß-naphthylamide (PAßN) in conjunction with amikacin, ceftazidime, imipenem, meropenem and levofloxacin. A search was conducted for structural genes that are linked to efflux pumps, and the relative expression of the *adeR*, *adeS*, and *adeL* genes was analyzed.

**Results:**

Among a total of 199 strains, 186 exhibited multidrug resistance (MDR). Fluoroquinolones demonstrated the highest resistance rates, while minocycline and amikacin displayed comparatively reduced resistance rates (1.5 and 28.1, respectively). The efflux activity of fluorquinolones exhibited the highest phenotypic detection (from 85 to 100%), while IMP demonstrated the lowest activity of 27% with PAßN and 43.3% with CCCP. Overexpression was observed in *adeS* and *adeL*, with *adeR* exhibiting overexpression.

Concluding that clinical strains of *A. baumannii* from our institution exhibited efflux pumps as one of the resistance mechanisms.

## Introduction

More than 10 million fatalities are anticipated to be attributed to antimicrobial resistance by 2050 [1, 2]. The pathogens comprising the ESKAPE group (*Enterococcus faecium*, *Staphylococcus aureus*, *Klebsiella pneumoniae*, *Acinetobacter baumannii*, *Pseudomonas aeruginosa*, and *Enterobacter spp*.) are strongly associated with a higher mortality risk due to their multidrug resistance [3, 4]. *A. baumannii* is a member of the *Moraxellaceae* family and belongs to the *Acinetobacter calcoaceticus baumannii* complex as well as *A. calcoaceticus*, *A. baumannii*, *A. pittii*, *A. nosocomialis*, *A. lactucae*, and *A. seifertii* [5, 6].

*A. baumannii* is a strictly aerobic, nonfermenting, nonfastidious, nonmotile, catalase positive, oxidase negative, and indole negative Gram-negative coccobacillus [7]. This bacterium has become a significant opportunistic pathogen in humans; annual infections have increased, and it is associated with high rates of resistance to multiple antibiotics; thus, the World Health Organization (WHO) has classified it as a critical priority bacterium since 2017 [8, 9]. *A. baumannii* is primarily linked to healthcare-associated infections, which manifest in various clinical forms including bacteremia, pneumonia, meningitis, urinary tract infection, and wound infection [10], besides is resistant to multiple drugs, and is associated with 7,300 infections and 500 fatalities annually in the United States (US), according to the Centers for Disease Control (CDC) and Prevention. Moreover, the global incidence of the disease is estimated to be around one million, and mortality rates vary between 25 and 61.6%, contingent upon the specific empirical treatment implemented [11, 12]. *A. baumannii* is capable to survive in harsh and stress pressure conditions such as dehydrated environments [13]. Besides, it bacterium may form biofilms over the wounds provoking complications in the care and treatment of burn patients [14]. Also, the prolonged hospitalization and the application of surgeries for treating and curing burn wounds may cause bacterial disseminations and generate bloodstream infections [15], therefore the care of this wounds is very delicate and critical. In the period of 2012–2015, the incidence of *A. baumannii* in infections of burn wounds reported in American hospitals was registered in around 1.2 cases by 100,000 persons, moreover, to find resistance to carbapenems in the isolates [16]. *A. baumannii* is reported in acquires resistance to antimicrobials [8, 17–20]. ß-lactamases, carbapenemases (KPC-2, KPC-3, KPC-10, IMP, OXA-23-like, and OXA-40-like) [21], aminoglycoside-modifying enzymes, target modification, changes in permeability, and efflux pump overexpression are the primary mechanisms implicated [22–25]. Antimicrobial resistance in *A. baumannii* can be associated with four types of efflux pumps: the major facilitator superfamily (MFS), the resistance-nodulation-division (RND) superfamily, the small multidrug resistance transporters (SMR), and the multidrug and toxic compound extrusion family (MATE) [22]. Currently, RND and MFS are the most described. The RND consists of the outer membrane factors (AdeC), periplasmic membrane fusion proteins (AdeA), and a complex formed by an inner membrane protein and a transporter (AdeB) [21–26]. Overexpression of efflux pumps in clinical MDR strains is the result of numerous single mutations in a two-component regulatory system (AdeRS) [21–27]. The AdeABC efflux pump, an RND, has been associated with decreased susceptibility to several antibiotics, such as aminoglycosides, tetracyclines, macrolides, chloramphenicol, trimethoprim, fluoroquinolones, some ß-lactams, and ethidium bromide [26, 28–32]. Principal MFS pumps in *A. baumannii* are TetA and TetB. Overexpression of TetA confers resistance to tetracycline, while TetB is associated with resistance to minocycline and tetracycline, meanwhile AbaF, another MFS, confers resistance to fosfomycin [29, 33]. Resistance to lipophilic cations and quaternary compounds is a characteristic feature of SMR family proteins found in bacteria and archaea. AbeS (***A****.* **b**aumannii **e**fflux pump of **S**MR family) shows a decreased susceptibility to several dyes, antimicrobials, and detergents [29, 34, 35]. ABC transporters are ATP-dependent proteins that export or import solutes [29, 35, 36]. The tripartite MacA-MacB-TolC transporter in *A. baumannii* mediates the extrusion of macrolides, peptide toxins, virulence factors, siderophores, lipopolysaccharides, and protoporphyrins [29, 35, 37–44]. On the other hand, MATE transporters are prevalent in bacteria as well as in advanced animals and plants. These efflux pumps can excrete cationic dyes, fluoroquinolones, and aminoglycosides in *A. baumannii*. Constituting a member of this bacterial family, AbeM has the capability to significantly elevate the minimal inhibitory concentration (MIC) of rhodamine 6G, ciprofloxacin, norfloxacin, gentamicin, triclosan, daunorubicin, ethidium bromide, and kanamycin by four folds. Additionally, it can increase the MICs of erythromycin, trimethoprim, tetraphenylphosphonium, and chloramphenicol by two fold [29, 35, 45].

However, MDR is genetically associated with the upregulation of efflux pumps in *A. baumannii*, but its role in clinical isolates obtained from patients with burns is still inadequately understood. The aim of the present study was to assess the distribution of efflux pumps and their regulatory gene expression in clinical strains of *A. baumannii* isolated from burned patients.

## Materials & methods

### Clinical strains

We included 199 *A. baumannii* clinical strains previously isolated from burned patients treated at the Centro Nacional de Investigación y Atención de Quemados (CENIAQ) from the Instituto Nacional de Rehabilitación Luis Guillermo Ibarra Ibarra in Mexico City. Only one strain was chosen for each patient. Clinical strains were identified, such as *A. baumannii* complex with Vitek 2 Compact (Biomerieux, France) and *A. baumannii *sensu stricto with the amplification of the bla_*OXA-51*_ (specie-specific) gene [46].

### Susceptibility test

Susceptibilities were performed with the method of broth microdilution method with Müeller Hinton Broth supplemented with cations and the following antibiotics tested: amikacin (AK), ampicillin/sulbactam (SAM), ceftazidime (CAZ), cefepime (FEP), ciprofloxacin (CIP), colistin (COL), doripenem (DOR), gentamicin (GEN), imipenem (IMP), levofloxacin (LVX), meropenem (MEM), minocycline (MIN), piperacillin/tazobactam (TZP), and tigecycline (TGC). Serial double dilutions were performed for each antibiotic, for AK, CAZ, FEP, CIP, COL, DOR, GEN, IMP, LVX, MEM, MIN and TGC were tested from 64 µg/mL to 0.062 µg/mL in each well of 96 wells. For SAM was tested from 32/16 µg/mL to 0.031/0.015, meanwhile for TZP was tested from 128/4 µg/mL to 0.125/4 µg/mL. The wells from line 12 are for growth control of each strain. Each well was loaded with 100 µL of each antibiotic and concentration before mentioned. And as inoculed with a final concentration of 5 × 10^4^ CFU/mL. *Pseudomonas aeruginosa* ATCC 27853 and *E. coli* ATCC 25922 were used as quality controls, as well the strain type *E.* coli ATCC 35218 was also included to evaluate only ß-lactams and ß-lactamase inhibitors. The procedure was conducted according to describe in manual M07-A10 (Methods for dilution antimicrobial susceptibility tests for bacteria that grow aerobically) [47]. The breakpoint used were those published in the manual M100 to determine the minimal inhibitory concentrations [48]. MIC was defined as the last concentration where growth was not observed.

### Pulsed field gel electrophoresis patterns

We performed pulsed gel electrophoresis (PFGE) to identify clonal patterns among clinical strains. Clinical strains were inoculated onto 5% blood sheep agar and incubated at 37° C for 18 h. Then, a single colony was propagated in Brain Heart Infusion broth (BD, USA) for 16 h at 37 °C. Plugs were prepared, lysed, and deproteinized. Digestion in agarose plugs was performed with 30 U of the restriction enzyme *Apa*I. Once digested, plugs were loaded into a 1% certified agarose (BioRad, USA) gel. Gels immersed in 0.5X TBE (1 M Tris base, 1 M boric acid, and 20 mM EDTA) were run at 14° C in a CHEF Mapper (BioRad, USA) with the following protocol: initial switch 5.3 seg, final switch 34.5 seg, at an angle of 120°, 6 V/cm, and a run time of 19.5 h. Gels were stained with 0.5X TBE and ethidium bromide (1 μg/mL) for 30 min and washed with 0.5X TBE for 45 min. Lambda PFGE ladder was used as a molecular weight marker (N0341S, New England BioLabs, USA) [49]. Images were captured with the Gel Doc XR + Gel Documentation System (BioRad, USA). The fingerprinting profiles were analyzed using the BioNumerics v.7.1 (Applied Maths, Belgium) software package. Typing of fingerprint profiles was carried out using the Dice similarity coefficient [50] and the Unweighted Pair Group Method with Arithmetic Mean [51] according to average linkage clustering methods. To reduce selection bias due to an identical clonal origin, a single strain was randomly chosen from each clonal pattern for further experimentation [52].

### Phenotypic assays with efflux pumps inhibitors

To evaluate the efflux pump activity against antibiotics we performed assays with efflux inhibitor and without, in order to observe differences between them. Carbonyl cyanide 3-chlorophenylhydrazone (CCCP, Sigma Aldrich, USA) was used as an uncoupler of phosphorylation [53] to disrupt the ionic gradient of bacterial membranes. On the other hand phenylalanine-arginine ß-naphthylamide (PAßN, Sigma Aldrich, USA), a peptidomimetic compound that competes with RND substrate [54], was also used. First, the concentrations that do not inhibit the bacterial growth were tested for both using Müeller-Hinton supplemented with cations were determined. Three concentrations of each efflux pump inhibitors (EPIs) (25, 50, and 100 μg/mL) were tested. Once the appropriate concentration was chosen, these EPIs were tested in combination with the following antibiotics: AK, CAZ, IMP, MEM, and LVX in a concentration range of 0.25 to 128 µg/mL; antibiotics without inhibitor were also tested. For CCCP and PAßN, the final concentrations tested were 50 μg/mL. *A. baumannii* ATCC 19606 [12] was used for quality control. An inhibitory effect, defined as EPI, was considered if the MIC value with an inhibitor decreased 3 folds compared to MIC values without an inhibitor [55–57]. These experiments were done in triplicate.

### Efflux pumps gene amplification

The structural genes for efflux pumps in *A. baumannii* were identified using PCR. For the AdeABC system, *adeA, adeB, and* two component system genes (*adeR* and *adeS)* were amplified, while *adeL* was amplified as representative for AdeFGH,. For the SMR, efflux pump *abeS* was selected; for MATE, *abeM;* and *tetA* and *tetB* for MFS members. One colony of each strain was used to extract DNA (Table 1) using Chelex® resin (BioRad, USA) and incubated at 96° C for 20 min, then tubes were centrifuged at 10,000 rpm, and the supernatants were separated into new tubes. The PCR conditions were the following: 10X buffer (Applied Biosystems, USA), 1 mM MgCl_2_, 2 mM each dNTP´s (Invitrogen, USA), and 10 pmol of each primer for every target. 1.5 U *Taq* polymerase (Invitrogen, USA) and 5 µL DNA. The final reaction volume was 50 µL of water free of DNAses. Amplification conditions were: 1 cycle at 5 min/95 °C, 35 cycles (50 s/95 °C, 60 s/56 °C, and 50 s/72 °C), 50 s/72 °C final extension. Amplicons were visualized in a 1% agarose gel and run at 100 V for 1 h. *A. baumannii* ATCC 19606 was used as a positive control.

**Table 1:** Primers sequences used for amplifications of genes associated with efflux pumps. RND, Resistance nodulation cell division; MATE, multidrug and toxic compound extrusion; SMR, small multidrug resistance; MFS, major facilitator superfamily

Efflux family	Operon	Gen	Primers used 5´-3	Amplicon size (bp)	Reference
RND	*AdeABC*	*adeA*	ADEA-F: CTCTAGCCGATGTCGCTCAA ADEA-R: ATACCTGAGGCTCGCCACTG	510	Gholami, et al*.* [52]
		*adeB*	ADEB-F: AAGTATGAATTGATGCTGC ADEB-R: AACGATTATATTGTTGTGG	860	Gholami, et al*.* [52]
		*adeR*	adeR-F: GGCATGAGTGTTATTCGG adeR-R: CTCAGAGTGTATATAAACGC	152	Atasoy, et al*.* [53]
		*adeS*	adeS-F: TCGCAAAGCGTTTTATTGTG adeS-R: CGATTTTTGACGGAAACCTC	164	Kuo H Y et al*.* [54]
	*AdeFGH*	*adeL*	adeL-F: AGGAGTGTGCGTGTGGATC adeL-R: GAAATCGGCATCGGTGCTG	146	Coyne S et al*.* [55]
MATE	-	*abeM*	abeM-F: GCTATTCCGAAGCATTAGGC abem-R: CCAAAGCAGGTATTGGTCCT	346	Kuo H Y et al*.* [54]
SMR	-	*abeS*	abeS-F: TAGAGAATTCATGTCTTATCTTTATTTAGC abeS-R: CGCTCTGCAGTTATAGATGGGTGTTTTTAG	465	Srinivasan et al*.* [29]
MFS	-	*tetA*	tetA-F: GTAATTCTGAGCACTGTCGC tetA-R: CTGCCTGGACAACATTGCTT	286	Sáenz et al*.* [56]
		*tetB*	tetB-F: TTGGTTAGGGGCAAGTTTTG tetB-R: GTAATGGGCCAATAACACCG	325	Yuan Q B et al*.* [57]
-	-	*16S*	16 s-F: GTAGCTTGCTACTGGACCTAG 16 s-R: CATACTCTAGCTCACCAGTATCG	154	Kuo H Y et al*.* [54]

### Relative expression for efflux pumps genes

Relative expression was determined to define if genes are overexpressed because they can confer resistance to antibiotics. Genes *adeR, adeS,* and *adeL* where selected because these can confer resistance if they are overexpressed and are associated to AdeABC and AdeFGH system. First, ARN was extracted from a bacterial suspension with PBS, then centrifuged at 5000 rpm/2 min, supernatant was removed, and then 1 mL Trizol was added. The pellet was vortexed for 60 s., and 200 µL chloroform was added to each tube. The tubes were vortexed again for 60 s and then incubated at room temperature for 30 min. After that, tubes were centrifuged at 12,000 rpm for 25 min, aqueous phase was taken and transferred to a new tube, to which 500 µL isopropanol was added. The tubes were gently mixed, incubated at room temperature for 15 min and then centrifuged at 12,000 rpm for 10 min. The supernatant was removed, and immediately, 500 µL of 75% (v/v) ethanol was added. The pellet was gently mixed, and another centrifugation step was done (10,000 rpm/10 min), alcohol was removed, and each tube was dried until all the alcohol evaporated. The pellet was hydrated, and RNA was quantified with NanoDrop (Thermo, USA) [58, 59].

Relative expression was quantified for genes mentioned above and were normalized with the 16S rRNA gene as housekeeping (Table 1), and RT-PCR reactions were performed with SuperScript with Taq platinum (Invitrogen, USA). The absence of DNA was corroborated, RNA was quantified at 260 nm with NanoDrop (Thermo, USA) for each sample, cDNA concentration was standardized at 40 ng, and PCR was performed using SYBR Green master mix. These experiments were done in triplicate. The expression analysis was done with ΔΔCt- method [60].

### Statistical analysis

Descriptive statistics are presented as absolute and relative frequencies for qualitative variables and the distribution of quantitative data was assessed for normality with the Shapiro–Wilk test. Mann Whitney U test was performed to compare relative expression between genes, and then statistical inferences were adjusted with the Bonferroni method. All tests were performed assuming a two-tailed hypothesis, and a p-value < 0.05 was considered statistically significant. Software STATA17 and GraphPad Prism 7 were used.

## Results

**Graph 1:**
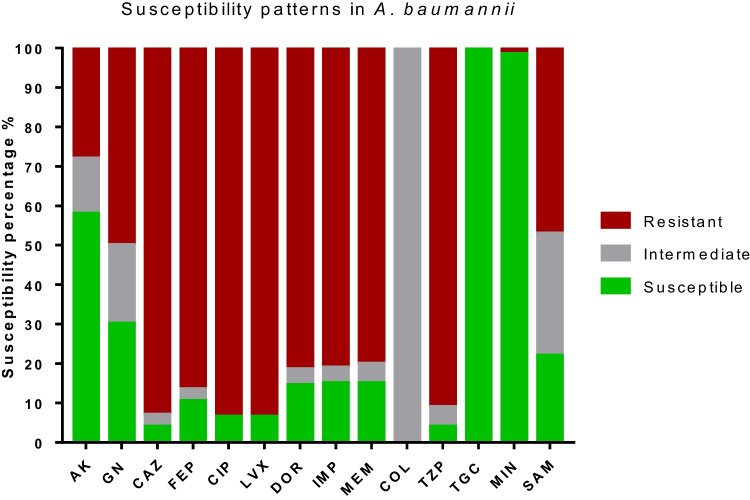
Susceptibility patterns of 199 A. baumannii strains isolated from burned patients. AK: amikacin, GEN: gentamicin, CAZ: ceftazidime, FEP: cefepime, CIP: ciprofloxacin, LVX: levofloxacin, DOR: doripenem, IPM: imipenem, MEM: meropenem, CST: colistin, TZP: piperacillin/tazobactam, TGC: tigecycline, MIN: minocycline and SAM: ampicillin/sulbactam

The microbiological characteristics and susceptibility patterns of clinical strains of *A. baumannii* were examined in this study. One-hundred and eighty-six strains (93.4%) of the 199 isolates obtained from patients with burns were MDR, while 13 strains (6.5%) were susceptible. The resistance rates to fluoroquinolones (CIP and LVX, 93.5%) were found to be the highest, followed by cephalosporins (CAZ and FEP, 93% and 86.5%, respectively) and TZP (91%). The resistance rates of AK (28.1%) and MIN (1.5%) were found the lowest. All strains were intermediate to COL and susceptible to TGC (Graph 1).

### Macrorestriction patterns in A. baumannii

**Fig. 1:**
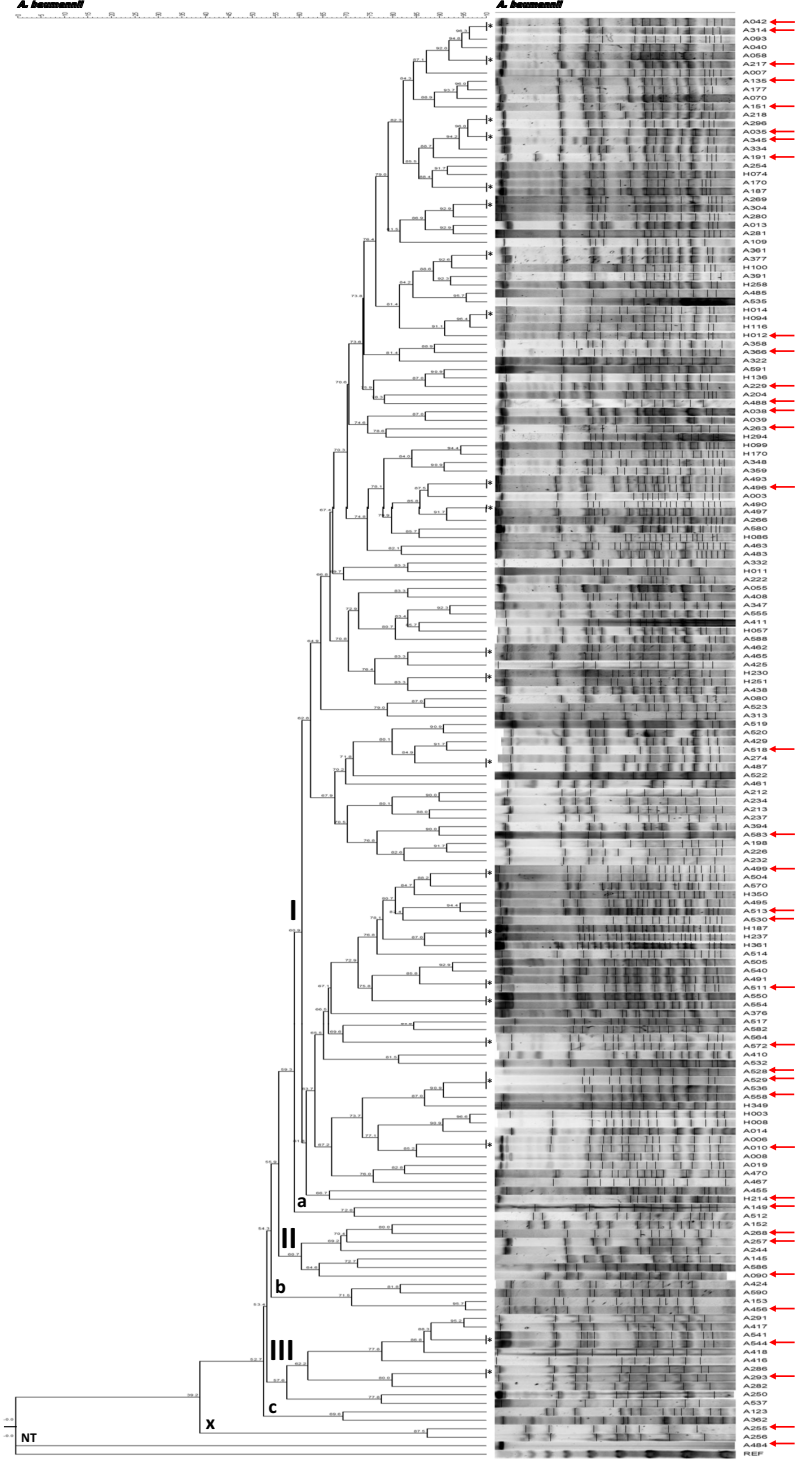
Dendrogram representing restriction patterns of *Acinetobacter baumannii* strains

Estimates of similarity among the 199 strains of *Acinetobacter baumannii* were summarized in the dendrogram presented in Fig. 1. The greatest part of the strains were members of three major divisions (I, II and III). In addition, there were three lineages (a, b, and c). Each of these lineages included two or four strains. The last branch from the major divisions was an *A. baumannii* strain that was not typable by PFGE.

The majority strains were in division I, in which there were thirty clusters, each consisting of a single strain or of a group of related strains.

Division II consists of seven strains, each of which was represented by a single chromosomal profile and division III comprise nine different profiles, two of which have two or more clonal strains.

The chromosomal profiles with an * (Fig. 1) include two or more strains from different samples. Strains included in the study are those with red arrows.

### Phenotypic assay with inhibitor of efflux pumps

After the strains exhibiting distinct clonal patterns were identified, one strain was selected for each group. For the groups comprised a minimum of two strains, only one strain per group was selected randomly, resulting in a final complement of 37 strains.

Phenotypic detection revealed that efflux pump activity was highest for fluoroquinolones (97.3%), followed by CAZ for PAßN (86.5%) and CCCP (89%). AK exhibited a 46% of each inhibitor utilized, while GEN demonstrated comparable results at 65%.

**Graph 2:**
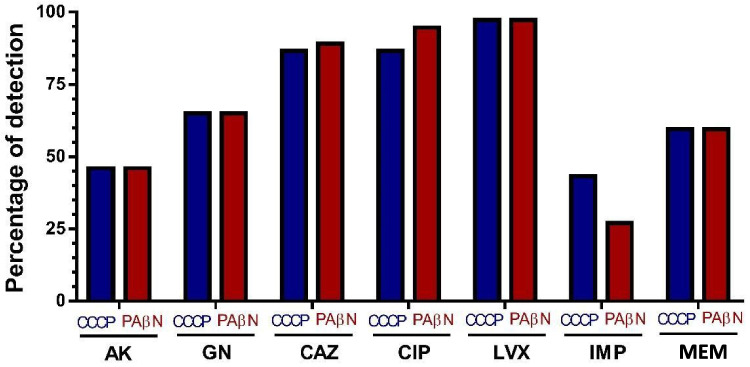
Resistance mediated by efflux pumps. Use of inhibitor and expressed such as percentage of detection. AK: amikacin, GN: gentamicin, CAZ: ceftazidime, CIP: ciprofloxacin, LVX: levofloxacin, IMI: Imipenem, MEM: meropenem, CCCP: carbonyl cyanide 3-chlorophenylhydrazone and PAβN: phenylalanine-arginine β—naphthylamide

Efflux pump-mediated resistance to MEM was identified in 59.5% of clinical strains; resistance mediated by efflux pumps to IMP was observed in 43.3% and 27% when CCCP or PAßN were used as inhibitors respectively (Graph 2).

### Amplification of genes associated to efflux pumps

The presence of the regulator *adeR* was detected in 94.6% of clinical strains, while *adeS* was detected in 92%. AdeABC was detected in 95% of all clinical strains examined by *adeB*, while *adeA* was present in 87%. However, a gene regulator associated with AdeFGH was detected in 22/37 (59.4%) of the clinical strains examined; each of the before mentioned genes is a component of the RND system.

In contrast, for *abeM* (MATE), the codifying gene was detected in 86.5% of clinical *A. baumannii*.

**Graph 3:**
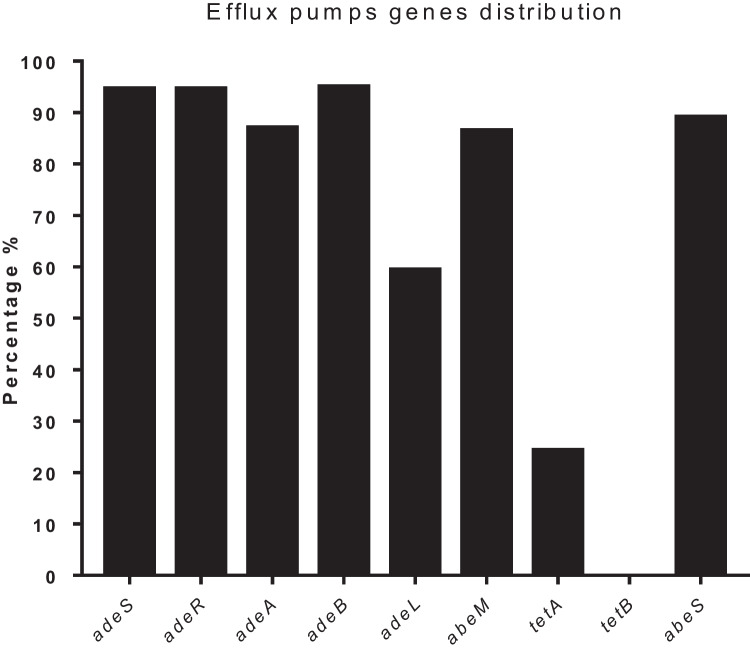
Distribution of efflux pumps present in A. baumannii

The resistance of *A. baumannii* to tetracyclines and MIN has been attributed to two efflux pumps belonging to the MFS family. Nevertheless, *tetB* remained undetected, and *tetA* was identified in a mere 24.3% of clinical strains. Gene *abeS*, which is linked to the SMR system, was identified in 89.1% of the clinical strains of *A. baumannii*, ranking as the second most prevalent gene (Graph 3).

### Relative expression of regulator genes of efflux pumps

**Fig. 2:**
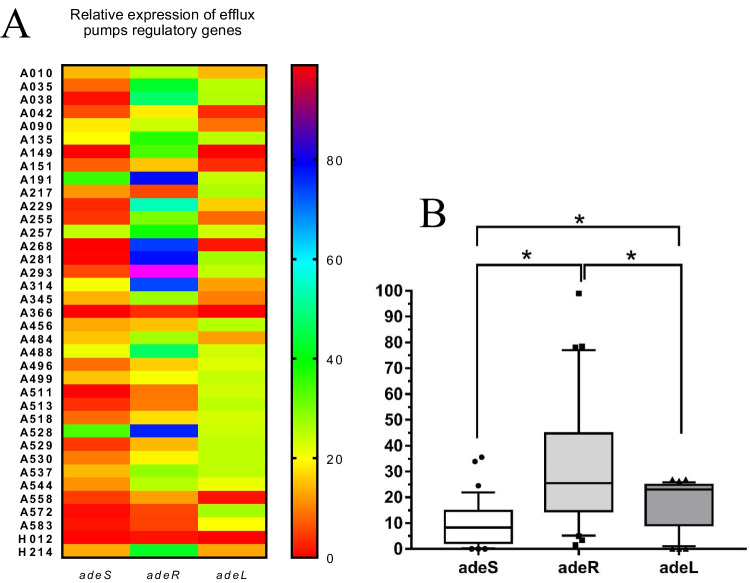
Relative quantification efflux pumps representation. **A**) represent in a heatmap expression levels in genes tested. **B**) box and whisker plots

Both *adeS* and *adeL* were identified as overexpressed RND genes in 35/37 and 36/37 strains, respectively. AdeR overexpression was observed in all 37 isolates. In accordance with the statistical significance demonstrated in Fig. 2, *adeR* exhibited the highest overexpression rate, followed by *adeL*. The lack of a Gaussian fit to the relative expression data was indicated by the W statistic and p-value (*adeS* W = 0.8967 & p = 0.0024; *adeR* W = 0.8896 & p = 0.0015; *adeL* W = 0.8172 & p < 0.001). Median, interquartile range (IQR), box and whisker diagrams, and so forth, were utilized to summarize the variables. Raw and adjusted p-values are provided (*adeR* vs. *adeS* [Mann–Whitney U = 246, p < 0.0001, adjusted p < 0.0003]; *adeL* vs. *adeR* [Mann–Whitney U = 442.5, p = 0.0084, adjusted p = 0.0252]; *adeL* vs. *adeS* [Mann–Whitney U = 384, p = 0.0010, adjusted p = 0.0030]).

## Discussion

Burns are a significant global public health concern that, in conjunction with the emergence of multidrug-resistant microorganisms, complicates the management and eradication of these pathogens in burn victims. Consequently, infections in this population may contribute to elevated mortality and morbidity rates [61]. *A. baumannii* has been described to be one of the most prevalent microorganisms capable of colonizing and infecting burned patients, due to its adaptability to various environments, including hostile ones [62]. *A. baumannii* possesses an inherent propensity for rapidly acquiring antimicrobial resistance mechanisms. It has been identified as the fifth cause of nosocomial infection and the second organism linked to nosocomial infections in intensive care units in 2015, according to reports from Mexican health institutions [63]. Furthermore, epidemiological investigations conducted by our institute identified it as a primary pathogen associated with infections in patients who have suffered burns (data not published). These results align with reports from South America, where this bacteria is listed as the second and third most prevalent causative agent, respectively [64]. According to reports from China, it is also among the most prevalent microorganisms; in the United States, the CDC has identified and categorized it as a clinically and economically significant etiological agent [65, 66].

In our study, 93.47% of clinical isolates were MDR, with high MICs for the principal antibiotics active against *A. baumannii*, including cephalosporins, fluoroquinolones, carbapenems, and ß-lactams with ß-lactamase inhibitors. Multiple mechanisms of resistance could potentially be linked to these patterns. In this study, no strains were identified that exhibited extensive drug resistance (i.e., nonsusceptibility to at least one agent in all but two or fewer antimicrobial categories) or pandrug resistance. Notably, all strains demonstrated minimal resistance to MIN, total susceptibility to TGC, and intermediate resistance to CST, suggesting that those antibiotics are a potential therapeutic option for these bacteria.

MDR rates exhibit variability, spanning from 22.8% to 100%, in the manner shown by Bialvaei data [67].

While not the primary aim of the study, the presence of additional mechanisms like extended-spectrum ß-lactamases or carbapenemases could potentially contribute to resistance to cephalosporins and carbapenems. Nevertheless, it has been noted that these mechanisms are not widespread among *A. baumannii* [68]. It is noteworthy that *A. baumannii* is commonly associated with extended-spectrum ß-lactamases of the GES, PER, and VEB types.

As the second most prevalent bacterium in our burn unit, *A. baumannii* causes nosocomial infections (those affecting the pulmonary system, bloodstream, epidermis, and soft tissue, as well as the urinary tract and devices). *A. baumannii* is frequently found in intensive care units, where it is associated with nosocomial infections due to its ability to colonize the gastrointestinal tract or remain on surfaces for over a year [69, 70].

We identified that a significant proportion of isolates exhibited quinolone resistance. Efflux pump mediation accounted for only 24% of activity. Interestingly, the resistance mechanisms against this family comprise multiple mechanisms, including those associated with the *gyrA* and *parC* genes.

One of the resistance mechanisms described in *A. baumannii* is overexpression of AdeABC, which confers resistance to several antibiotic groups [71]. The use of CCCP and/or PAßN has been cited extensively in several publications. The inhibitors were employed in this study to phenotypically illustrate the existence and functionality of these pumps; however, the determination of the efflux pump type is exclusively possible by employing molecular tools [72]. The use of CCCP or PAßN did not show any difference in the detection of efflux pumps that confer resistance to aminoglycosides, LVX, and MEM. The PAßN demonstrated greater efficacy in identifying resistance associated with efflux pumps for CAZ and CIP, whereas the CCCP demonstrated greater effectiveness in inhibiting efflux pumps that confer resistance to IMP; however, the lack of porins must be taken into consideration.

To the best of our knowledge, this is the first study that, describes resistance associated with efflux pumps in *A. baumannii* strains isolated from burnt patients. Antibiotic resistance was observed to be associated with the presence of at least one type of efflux pump in the clinical strains that were examined. According with our results, we suggest that resistant to fluoroquinolones and CAZ mediated by efflux pump is an MDR mechanism predominant in bacterial infections observed in burn patients. In clinical strains of *A. baumannii*, the primary resistance mechanism linked to MDR was the upregulation of efflux pumps; the MDR phenotype is enhanced when this mechanism concurrently operates with the synthesis of carbapenemases. Although the MDR mechanism linked to efflux pump activity also might be associated with mutations in the AdeABC system [73, 74], or the presence of other types of efflux pumps such as RND, SMR or MSF, among others [29]. However, our study did not contemplate to perform studies for finding mutations in the components of efflux pumps or testing discriminatory inhibitory assays for evaluating the specific activity of other types of efflux pumps; indeed, we contemplate in a further study to reinforce the current data with more robust analysis by using genomic analysis [29]. Currently, there not exists treatment that specifically targets efflux transporters found in Gram-negative bacteria. The potential inhibition of efflux pumps is a significant consideration in the clinical practice of MDR infections; however, optimal drugs must be stable and non-toxic to eukaryotic cells. Various potential drugs have been reported to either interact as substrates (competitive inhibition) or directly influence the proton gradient (non-competitive inhibition) [75]. Potential drugs for inhibiting efflux pumps in Gram-negative bacteria could be verapamil, amlodipine, or reserpine, which have been evaluated previously in *A. baumannii* isolates [76–78], acting as calcium channel blockers in the AdeABC efflux system for amlodipine; MFS and RND as well for reserpine. However, these drugs have only been evaluated in vitro, and the main concern is associated with potential toxicity that may lead to the avoidance of their use. Nevertheless, despite of that currently there is not antibiotic for treating infections in burn wounds caused by *A. baumannii* targeted in inhibiting efflux pump activity; is necessary to encourage studies for the searching new molecules with antimicrobial activity targeted to the inhibition of efflux pumps.

## Conclusions

Clinical strains of *A. baumannii* from our institution exhibited efflux pumps as one of the resistance mechanisms, thereby possibly constraining therapeutic alternatives. Therefore, it is crucial to prioritize the exploration of natural, synthetic, or semisynthetic efflux pump inhibitors molecules to integrate them into conventional treatment approaches. We observed a substantial concurrence was noted between phenotypic and genotypic assays for determining the activity of efflux pumps; both methodologies enable the detection of their presence. Identifying these resistance mechanisms is of the utmost importance to prevent their proliferation within a hospital environment and to establish a surveillance program that can identify strategies to reduce pressure selection in these clinical strains.

## Data Availability

Raw data were generated. Derived data supporting the findings of this study are available from the corresponding author LELJ on request.
